# Lower limb body composition is associated to knee passive extension torque-angle response

**DOI:** 10.1186/2193-1801-2-403

**Published:** 2013-08-27

**Authors:** Tiago Neto, Sandro Freitas, João Vaz, Analiza M Silva, Pedro Mil-Homens, Ana Isabel Carita

**Affiliations:** Faculdade de Motricidade Humana, CIPER, Universidade de Lisboa, P-1100 Lisbon, Portugal

**Keywords:** Body composition, Flexibility, Passive stiffness, Viscoelastic stress relaxation, Anthropometry

## Abstract

**Purpose:**

People vary in flexibility regarding maximum joint angle, resistance to stretch and mechanical responses during stretching exercises. Body composition (BC) has been been mentioned as one of the factors for flexibility differences. The aim of this study was to determine how body composition and anthropometric measures of the lower limb is associated with passive knee extension (PKE) torque-angle (T-A) response.

**Methods:**

Twenty-five male subjects with poor flexibility performed a maximal PKE repetition (velocity of 2°/s; 90 seconds in the static phase). Knee passive T-A, vastus medialis and semitendinosous electromyographic activity were recorded during the protocol. Viscoelastic stress relaxation (VSR) amplitude, knee passive stiffness (KPS), lower limb body composition assessed by dual energy x-ray absorptiometry, and anthropometry measures were determined.

**Results:**

Thigh skeletal muscle and bone mass, as well as thigh perimeter, showed a moderated correlation with passive torque (r = 0.45; r = 0.6; r = 0.59, respectively), joint angle (r = 0.46; r = 0.5; r = 0.5), and VSR (r = 0.46; r = 0.49; r = 0.5). Thigh skeletal muscle was also correlated with KPS (r = 0.42). All these correlations were statistically significant (p < 0.05).

**Conclusions:**

Passive knee extension T-A was found to be moderately correlated with lower limb BC. In particular, thigh perimeter and skeletal muscle mass were associated with knee passive stiffness and viscoelastic stress relaxation. More research is needed to understand what influences joint maximum angle, resistance to stretch and mechanical response to stretching.

## Introduction

A number of studies have reported that joint passive torque-angle response varies among subjects (Alonso et al. 2009; Magnusson et al. 1995; McHugh et al. 1996). Consequently, different maximum joint angle, passive torque response, passive peak torque (PT) tolerated, viscoelastic stress relaxation (VSR) amplitude, and VSR time course are obtained (Klinge et al. 1997; Kubo et al. 2002; Magnusson et al. 1996a, 2000). Such variability can be seen by the standard deviation (SD) value of torque-angle outputs of different studies (Klinge et al. 1997; Magnusson et al. 1996a, 2000), since it is a measure of variability. In respect to joint PT, previous studies have reported a SD ranging from 11% to 27% of the peak torque value (Klinge et al. 1997; Magnusson et al. 1996a, 2000). The SD of VSR amplitude has varied from 7.5% to 25% of joint peak torque value (Klinge et al. 1997; Magnusson et al. 1996a, 2000).

The variability of torque-angle responses has been explained by several structural factors. For instance, Ryan et al. (2009) observed a positive correlation between leg muscles cross sectional area (CSA) and ankle dorsiflexion passive torque. Magnusson et al. (1997) also observed a positive correlation between thigh muscle CSA and knee extension passive torque. Gajdosik (2006) found that the electromyographic response (> 1% of maximal voluntary contraction) of the muscles being stretched affected VSR amplitude during the static phase. In addition, body composition (BC) has also been suggested to explain flexibility differences (Faria et al. 2009; Magnusson et al. 1997; Ramadan and Barac-Nieto 2003). However, the evidence explaining the relationship between BC and flexibility is scarce. Previous studies only have used body fat percentage through skinfold assessment (Ramadan and Barac-Nieto 2003) and body mass index (BMI) (Faria et al. 2009) to infer about flexibility differences. Although these BC measurements are easily performed and low-cost (Piers et al. 2000), they do not give information in relation to lean mass, or muscle mass. Using dual-energy x-ray absorptiometry (DXA) technology it is possible to collect valid and reliable data about bone mass, fat mass, and lean mass (De Lorenzo et al. 1998; Kitano et al. 2001). In fact, more precise and valid BC measures are obtained by DXA when compared to bioelectrical impedance analysis (Eisenkolbl et al. 2001; Gupta et al. 2011), or skinfold measures (Wattanapenpaiboon et al. 1998). In addition, this method has the advantage of performing faster analysis, is less expensive, and uses less radiation when compared to computerised tomography (Plank 2005). To our knowledge, no studies have used DXA measures to investigate joint passive torque angle response in both dynamic (when the joint is mobilized) and static (when the leg is in a stationary position) phases of a stretch procedure.

Knee passive extension torque-angle has been used in some studies for different purposes (Klinge et al., 1997; Magnusson et al. 1996a, 2000), but few have aimed to analyze the factors underlying the differences between subjects. The purpose of this study was to determine if BC, specifically of lower limb, could explain knee passive extension torque-angle differences in normal subjects. It was hypothesized that subjects with higher lower limb muscle mass would have a torque-angle curve with greater stiffness and increased VSR amplitude.

## Materials and methods

### Subjects

Twenty-five male subjects (21.8 ± 2.9 years, 73.9 ± 8.9 kg, 1.75 ± 0.1 m; mean ± SD), physically active, with less than 70° of active knee extension with the hip flexed to 90 degrees (assessed with a standard goniometer), and injury free to the lower limbs, participated in this cross-sectional study. Written informed consent was obtained from all participants, accordingly to the Helsinki Declaration on Human Experimentation. This research has been approved by the ethics Committee of the Faculty of Human Kinetics, University of Lisbon.

### Outcomes

#### Torque-angle

**Figure 1:**
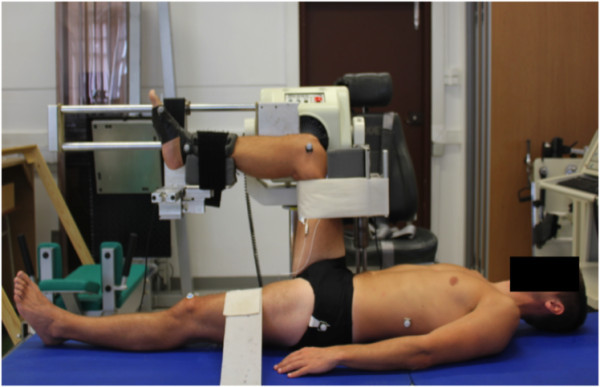
**Experimental setup for the knee extension angle-torque assessments.**

Knee passive extension torque-angle outcomes were determined in a reliable experimental setup (Figure 1), described in detail elsewhere (Freitas et al. unpublished observations). The right lower limb of all subjects was tested in this study. Knee PT was determined by measuring knee extension resistance to stretch at a specific leg point, and normalizing to its distance to knee axis (lateral femoral condyle). Knee extension resistance to stretch was measured by a device that fitted in a dynamometer (Biodex Model System 3), with a force sensor (load cell 1042, Sensor Techniques Ltd, UK) incorporated in a support connected to two shafts by a system of spheres that moved the subject’s leg. Knee angle was measured by the position output of Biodex. Force and position output were collected at 500 Hz. Subjects lay supine with the right hip flexed at 90°, the knee axis (lateral femoral condyle) aligned to the dynamometer shaft, and with the leg horizontally positioned over the device support that fitted to dynamometer, making 90° knee angle. The right ankle was immobilized in neutral position (90°) as well as the contralateral lower limb. Freitas et al. (unpublished observations) have shown previously that assessing force through a sensor closer to the limb being tested, and joint angle by 2D kinematic analysis had a higher reliability compared to conventional dynamometer assessment. In the present study, knee angle was assessed through dynamometer output, and force by the sensor incorporated in the device that moved the leg.

#### Body composition measurements

Body composition was assessed using DXA (Hologic Explorer-W, fan-beam densitometer, software QDR for Windows version 12.4; Hologic, Waltham, Massachusetts, USA). We performed total body scans, from which we retrieved total body fat, bone mass, and lean soft tissue mass. Using regional analysis, we were able to specify these BC components for thigh and leg segments. Shih et al. (2000) formula (skeletal muscle = 0.735 × lean regional mass + 0.38) was used to determine lower limb skeletal muscle. Afterwards, only BC for the right lower limb was used to compare with the flexibility values. Subjects were instructed to avoid any extenuating physical activity in the preceding 48 h. They also had three hour period with no food ingestion, and one hour period with no liquid ingestion prior to BC assessment.

#### Anthropometry measurements

Anthropometry assessment included weight, height, thigh length and perimeter, leg length and perimeter. These measures were performed accordingly to the International Society for the Advancement of Kinanthropometry guidelines. These measures had a high reliability value (r = .99).

#### Electromyography

In order to ensure a passive nature to the knee extension, surface electromyography (EMG) of vastus medialis (VM) and semitendinosous (ST) were measured. Active bipolar surface electrodes (Plux, Portugal), with a band-width of 20-450 Hz were used do record and amplify (gain of 1000 and a common mode rejection ratio > 120 dB) the EMG signals. EMG data were digitized, at a sampling rate of 500 Hz, together with torque and angle signals. All EMG signals were normalized to the Maximal voluntary isometric contraction (MVIC) values, and expressed as a percentage of this value. Details of the EMG procedures can be founded elsewhere (Freitas et al. unpublished observations).

### Data processing

Force, knee angle, and EMG data were synchronized and recorded, with a sampling rate of 500 Hz, using BIOPAC MP100 Acquisition System (Santa Barbara, USA). Data was subsequently processed by a specific designed automatic routine using the MATLAB® v12.0 software (The Mathworks Inc, Natick Massachusetts, USA), specifically explained in another investigation (Bruno et al. unpublished observations). In short, this routine first filtered the force data using a Butterworth second order low pass filter (12 Hz). Then, it determined knee passive torque by multiplying passive resistance to knee extension (F_p_) by the leg length (L_leg_). Torque was corrected to gravity data by subtracting the leg-foot-device weight (W_LFD_) using a simple cosine function (McHugh et al. 1992; Freitas et al. unpublished observations), through the follow mathematical expression: PT = F_P_ × L_Leg_ - cos (α_Knee_) × W_LFD_ × L_Leg_. The W_LFD_ was determined in the testing start position before the dynamic phase, by measuring the average force in one-second interval prior to the beginning of dynamic phase. Then, a specific mathematical model proposed by Bruno and colleagues was fitted to the torque-angle data for both dynamic and static phases (Bruno et al. unpublished observations). Briefly, dynamic phase was fitted with an exponential model given by the equation:1

where b_0_ is a parameter to be estimated, T_1_ is the last time of dynamic phase, and A_1_ is the true observed ordinate of T_1_. Static phase consisted in two different components (fast and slow) and was fitted with a biexponential model given by a combination of two functions, such that:2

where b_1_, b_2_, τ_1_ and τ_2_ are parameters to be estimated, T_1_ is the last time of dynamic phase, T_2_ is the last time of fast component in static phase, and A_i_ is the true observed ordinate of T_i_, i = 1, 2.

Additionally, the automatic routine also normalized the EMG activity of the muscles tested to the maximal EMG obtained in MVIC for knee extension and flexion, in all stretching repetitions.

### Procedures

Subjects came three times to the laboratory. Initially, a familiarization session with the experimental setup was performed, and instruction was given about the assessment session and the exercise limitation requirement in the previous 24 hours before the test. During this session, anthropometry was also collected. In the second visit, subjects performed one repetition of the knee passive extension with an angular velocity of 2°/s. They were instructed to say “OK” when they felt the maximum stretch without pain or discomfort. This amplitude was defined as the maximal range of motion. No warm-up was performed. Once maximal knee angle was reached, subjects held it for 90 seconds in the static position. After the protocol, subjects performed three MVIC for the knee flexors and extensors using a dynamometer, in order to determine the maximal EMG activity of VM and ST. For MVIC recordings subjects were seated with their knee angle at a 90°. In the third visit, the BC assessment through DXA was performed.

### Statistical analysis

Descriptive statistics were used to characterize the BC, anthropometric and flexibility variables. These consisted in knee extension angle, PT, and passive stiffness (estimated by dividing torque by angle at 50° and expressed in N·m/°), the absolute VSR (corresponding to the torque decline during the static phase), and the relative VSR (corresponding to the absolute VSR normalized to the peak torque). Normality was verified in all torque-angle data with Shapiro-Wilk test. Correlations using Pearson coefficient were used to see the relation between BC and anthropometric values, passive torque, knee angle, VSR and passive stiffness. Subjects were later divided in two groups (A and B) according to their BC values, and thigh anthropometry. Group A included 10 subjects with the lowest values of lean and bone mass, and also less thigh length and perimeter; Group B was also composed by 10 subjects, with the highest values in lean and bone mass, and thigh length and perimeter. Torque-angle variables were compared, between groups using Student’s T-test for independent samples. Statistical significance for all tests was set at p-value < 0.05.

## Results

EMG values recorded were below 3% of the MVIC. Descriptive torque-angle, anthropometric, and body composition data are presented in Table 1. Subjects produced a knee passive peak torque of 48.9 ± 15.8 N · m and a maximal angle of 67.8 ± 8.8°. Knee flexors passive stiffness was 0.56 ± 0.12 N · m/°. Mean absolute VSR was 9.45 ± 3.37 N · m, and mean VSR normalized to peak torque was 19.51 ± 4.95%.

**Table 1:** **Mean ± standard deviation of body composition, anthropometry, and torque-angle values, for total body, and thigh segment**

Variable	Total (n = 25)	Group A (n = 10)	Group B (n = 10)
**Total fat mass (kg)**	17.82 ± 19.5	10.97 ± 3.93	12.43 ± 4.03
Total bone mass (kg) *	2.74 ± 0.49	2.42 ± 0.5	3.01 ± 0.37
Total lean Mass (kg) *	61.55 ± 7.47	54.7 ± 6.57	67.44 ± 2.96
**Thigh fat mass (kg)**	1.76 ± 0.74	1.64 ± 0.76	1.82 ± 0.76
Thigh bone mass (kg) *	0.28 ± 0.05	0.24 ± 0.05	0.32 ± 0.04
Thigh skeletal muscle (kg) *	5.67 ± 0.77	4.93 ± 0.58	6.36 ± 0.29
Thigh length (cm) *	38.47 ± 2.83	37.05 ± 3.42	40.32 ± 1.58
Thigh perimeter (cm) *	53.0 ± 3.66	50.83 ± 4.56	54.49 ± 2.15
**Passive Torque (N · m)**	48.9 ± 15.8	41.43 ± 15.38	52.6 ± 16.51
**Angle (°)**	67.8 ± 8.8	65.74 ± 8.30	68.55 ± 8.21
**Stiffness (N · m/°)**	0.56 ± 0.12	0.49 ± 0.09	0.57 ± 0.08
Absolute VSR (N · m) *	9.45 ± 3.37	7.29 ± 3.03	10.15 ± 2.62
**Relative VSR (%)**	19.51 ± 4.95	17.45 ± 3,22	19.82 ± 3,61

* - Significantly different between group A and B (p < 0.05).

*VSR* Viscoelastic stress relaxation.

**Figure 2:**
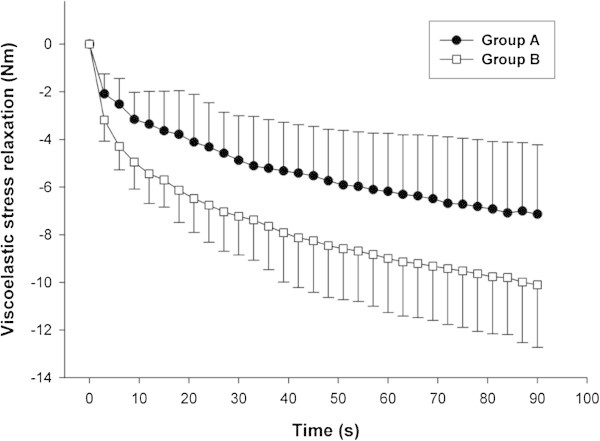
**Viscoelastic stress relaxation observed during the static phase.** Detailed legend: Absolute viscoelastic stress relaxation was different (p = 0.037) between group A (7.29 N · m) and group B (10.15 N · m).

Total and regional BC and anthropometric measures are presented in Table 1. Significant differences were observed between group A and B in all measures, with the exception of total and thigh fat mass. Individuals from group A had lower values in all flexibility variables. However, only absolute VSR was significantly different between groups (p = 0.037) (Figure 2).

**Figure 3:**
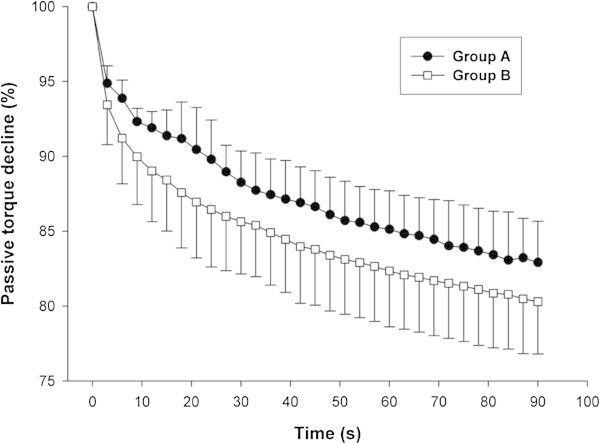
**Passive torque decline normalized to the initial peak torque (relative viscoelastic stress relaxation).** Detailed legend: There was a difference in torque percent decline between groups, in 6 s - 30 s period. From second 30 until the end of the static phase, there were no differences between groups.

In Figure 3, the passive torque decline normalized to the initial peak torque, throughout the static phase. Subjects from group B had a greater decline in passive torque between second 6 and second 30. During the remaining time, this difference was not statistically significant.

Pearson correlation coefficients are presented in Table 2. The highest correlations were found between PT and thigh perimeter, thigh bone mass, and BMI. VSR was significantly correlated to thigh perimeter, thigh bone mass, and thigh skeletal muscle. Passive stiffness was only correlated to thigh skeletal muscle.

**Table 2:** **Pearson correlation values between angle-torque variables and body composition and anthropometry values**

	Thigh perimeter	Thigh bone mass	Thigh skeletal muscle	BMI
**Passive torque**	0.59*	0.60*	0.45*	0.59*
**Angle**	0.50*	0.46*	0.22	0.55*
**VSR**	0.50*	0.46*	0.49*	0.41*
**Stiffness**	0.37	0.18	0.42*	0.28

* - Significant correlation (P < 0.05).

Legend: *VSR* Viscoelastic stress relaxation, *BMI* Body mass index.

## Discussion

The results of this study showed a moderate and significant correlation between BC, anthropometry, and the individual’s flexibility. Specifically, thigh bone mass, thigh skeletal muscle, and thigh perimeter, were all correlated to passive torque and VSR.

The values of peak torque, knee angle, stiffness, and VSR reported in this study, are in accordance with other investigations. Magnusson et al. (1996a) and Klinge et al. (1997) also reached similar values in a knee passive extension test. Only maximal knee angle was different and that can be explained by the testing protocol, in which angle was quantified with the subjects seated and the hip flexed at 30° to a horizontal line. This position represents an additional elongation to the knee flexors, and consequently the knee extension angle was lower. Regarding VSR, we observed a global 20% torque reduction from peak torque through the 90 seconds of the static phase. This value is slightly lower than some investigations (Klinge et al. 1997; Magnusson et al. 1996a) that also used 90 seconds in the static phase, but similar to Magnusson´s study that only used 45 seconds (Magnusson et al. 2000).

We initially hypothesized that the correlation between the values of BC components (fat mass and lean mass) and the flexibility parameters would be higher than those actually obtained. In comparison, Ryan et al. (2009) observed a correlation of r = 0.83 between muscle CSA and plantar flexors passive stiffness. Nevertheless, the muscle group analyzed by Ryan et al. (2009) investigation was different from our investigation, which might give some insight regarding the difference in the *r* values. Moreover, data from the current study indicates that the leg segment has nearly half of the thigh fat mass, which might also influence the correlation magnitude. Using a protocol targeting the knee flexors, Magnusson et al. (1997) reached a correlation of r = 0.66 between hamstrings CSA and passive stiffness. Our investigation revealed a smaller correlation (r = 0.42) between thigh muscle mass and the knee flexors passive stiffness. The reason for such difference might be related to the fact that DXA does not allow a clear distinction between knee flexors skeletal muscle from knee extensors, in comparison to magnetic resonance imagining or peripheral quantitative computed tomography, as used in Magnusson et al. (1997) and Ryan et al. (2009). In addition, we observed that BMI had nearly the same correlation with passive torque and VSR than thigh skeletal muscle or thigh perimeter. Faria et al. (2009) also found a moderate correlation (r = 0.48) between BMI and plantar flexors passive stiffness. This leads us to conclude that, despite the fact that two different muscle groups were assessed (plantar flexors and knee flexors), the correlation level with passive stiffness is similar by either using BMI or body segment skeletal muscle determined by DXA.

In respect to static phase response, our results have shown that subjects with higher thigh skeletal muscle, and larger thigh perimeter, had a greater absolute VSR. However, when normalized to peak torque this difference was smaller and non-significant. Even though, during the first 30 seconds of static stretching there was a significant increase in the passive torque decline of subjects with larger skeletal muscle mass, this can be related to the fact that these subjects need less time to complete the fast VSR component. Several investigations have studied viscoelastic stress relaxation, trying to characterize and to understand what influences the stress relaxation phenomenon (Duong et al. 2001; Magnusson et al. 1995, 1996b). One explanation often suggested for this phenomenon is related to the connective tissue mechanical response (Purslow et al. 1998; Thorton et al. 2001). In particular, collagen fibers orientation has been put forward as one of the mechanisms for stress relaxation (Mijailovich et al. 1993). However, Purslow et al. (1998) did not observed a large-scale reorientation of collagen fibers during the stress relaxation time course. Thus, this phenomenon might not be the one responsible for VSR. Another explanation advanced for the VSR may be related to connective tissue hydration changes due to a long enough static stretch (Schleip et al. 2012; Thorton et al. 2001). In a recent study of Schleip et al. (2012), it was observed an immediate fluid content decrease that accompanied the VSR of a rat lombodorsal fascia placed under static stretch by 15 minutes. The fact that higher skeletal muscle mass carries more water content, might explain why BC is associated with VRS response. Another element also suggested to be related with VSR is the muscle EMG activity over the stretching period (Gajdosik 2006). Usually, this is a well-controlled parameter in these type of investigations in order to ensure a passive nature of the movement, and consequently having no considerable EMG activity. Researchers normally define an EMG activity below 1% (Kubo et al. 2002; Magnusson et al. 1996a; Ryan et al. 2009), or even 2% of the MVIC (Mizuno et al. 2013). Gajdosik (2006) recently addressed this question and found that low-level surface EMG (> 1% of MVIC) in the soleus and gastrocnemius muscles may influence the VSR process in aged population. Our mean surface EMG values (< 3% of MVIC) were higher than those reported by Gajdosik (2006), which might indicate an influence in the VSR phenomenon of some individuals. However we must consider the differences between these two investigations. We used a younger population, and monitored the knee flexors, instead of the plantar flexors. It is possible that EMG records suffer higher variations in the knee flexors, than in the plantar flexors. Blazevich et al (2012) reached, in their study, even higher EMG values, in the order of 8,5% for the soleus muscle, and 5,5% for the medial gastrocnemius.

In addition to the BC profile, the electromyographic activity, together with the tissues hydration status, and the possible relaxation process occurring in the matrix surrounding collagen fibers (Purslow et al. 1998), might give a plausible explanation for the VSR, attested by the time-dependent torque decrease in a static angle.

## Conclusions

Some of the key reasons for the differences in flexibility among normal subjects has not been clarified in previous studies. The results of this study demonstrate that torque-angle response is partially associated with body composition. Specifically, thigh muscle mass, and thigh bone mass, as well as thigh perimeter, were all moderately correlated to peak torque and viscoelastic stress relaxation. However, flexibility differences cannot be explained alone by anthropometric and body composition. Future research should address the importance that intrinsic tissues properties may have in subject’s flexibility differences.

## References

[CR1] Alonso J, McHugh MP, Mullaney MJ, Tyler TF (2009). Effect of hamstring flexibility on isometric knee flexion angle-torque relationship. Scand J Med Sci Sports.

[CR2] Blazevich AJ, Cannavan D, Waugh CM, Fath F, Miller SC, Kay AD (2012). Neuromuscular factors influencing the maximum stretch limit of the human plantar flexors. J Appl Physiol.

[CR3] De Lorenzo A, Bertini I, Candeloro N, Iacopino L, Andreoli A, Van Loan MD (1998). Comparison of different techniques to measure body composition in moderately active adolescents. Br J Sports Med.

[CR4] Duong B, Low M, Moseley AM, Lee RY, Herbert RD (2001). Time course of stress relaxation and recovery in human ankles. Clin Biomech.

[CR5] Eisenkolbl J, Kartasurya M, Widhalm K (2001). Underestimation of percentage fat mass measured by bioelectrical impedance analysis compared to dual energy X-ray absorptiometry method in obese children. Eur J Clin Nutr.

[CR6] Faria A, Gabriel R, Abrantes J, Brás R, Moreira H (2009). Triceps-surae musculotendinous stiffness: Relative differences between obese and non-obese postmenopausal women. Clin Biomech.

[CR7] Gajdosik RL (2006). Influence of a low-level contractile response from the soleus, gastrocnemius and tibialis anterior muscles on viscoelastic stress-relaxation of aged human calf muscle-tendon units. Eur J Appl Physiol.

[CR8] Gupta N, Balasekaran G, Govindaswamy V, Hwa C, Shun L (2011). Comparison of body composition with bioelectric impedance (BIA) and dual energy X-ray absorptiometry (DEXA) among Singapore Chinese. J Sci Med Sport.

[CR9] Kitano T, Kitano N, Inomoto T, Futatsuka M (2001). Evaluation of body composition using dual-energy X-ray absorptiometry, skinfold thickness and bioelectrical impedance analysis in Japanese female college students. J Nutr Sci Vitaminol (Tokyo).

[CR10] Klinge K, Magnusson S, Simonsen E, Aagaard P, Klausen K, Kjaer M (1997). The effect of strength and flexibility training on skeletal muscle electromyographic activity, stiffness, and viscoelastic stress relaxation response. Am J Sports Med.

[CR11] Kubo K, Kanehisa H, Fukunaga T (2002). Effect of stretching training on the viscoelastic properties of human tendon structures in vivo. J Appl Physiol.

[CR12] Magnusson S, Simonsen E, Aagaard P, Gleim G, McHugh M, Kjaer M (1995). Viscoelastic response to repeated static stretching in the human hamstring muscle. Scand J Med Sci Sports.

[CR13] Magnusson S, Simonsen E, Aagaard P, Kjaer M (1996). Biomechanical responses to repeated stretches in human hamstring muscle in vivo. Am J Sports Med.

[CR14] Magnusson S, Simonsen E, Dyhre-Poulsen P, Aagaard P, Mohr T, Kjaer M (1996). Viscoelastic stress relaxation during static stretch in human skeletal muscle in the absence of EMG activity. Scand J Med Sci Sports.

[CR15] Magnusson S, Simonsen E, Aagaard P, Boesen J, Johannsen F, Kjaer M (1997). Determinants of musculoskeletal flexibility: viscoelastic properties, cross-sectional area, EMG and stretch tolerance. Scand J Med Sci Sports.

[CR16] Magnusson S, Aagaard P, Nielson J (2000). Passive energy return after repeated stretches of the hamstring muscle-tendon unit. Med Sci Sports Exerc.

[CR17] McHugh M, Magnusson S, Gleim G, Nicholas J (1992). Viscoelastic stress relaxation in human skeletal muscle. Med Sci Sports Exerc.

[CR18] McHugh M, Kremenic I, Fox M, Gleim G (1996). The relationship of linear stiffness of human muscle to maximum joint range of motion. Med Sci Sports Exerc.

[CR19] Mijailovich S, Stamenovic D, Fredberg J (1993). Toward a kinetic theory of connective tissue micromechanics. J Appl Physiol.

[CR20] Mizuno T, Matsumoto Y, Umemura Y (2013). Viscoelasticity of the muscle-tendon unit is returned more rapidly than range of motion after stretching. Scand J Med Sci Sports.

[CR21] Piers LS, Soares MJ, Frandsen SL, O’Dea K (2000). Indirect estimates of body composition are useful for groups but unreliable in individuals. Int J Obes Relat Metab Disord.

[CR22] Plank L (2005). Dual-energy x-ray absorptiometry and body composition. Curr Opini Clin Nutr Metab Care.

[CR23] Purslow PP, Wess TJ, Hukins DW (1998). Collagen orientation and molecular spacing during creep and stress-relaxation in soft connective tissues. J Exp Biol.

[CR24] Ramadan J, Barac-Nieto M (2003). Reported frequency of physical activity, fitness, and fatness in Kuwait. Am J Hum Biol.

[CR25] Ryan ED, Herda TJ, Costa PB, Defreitas JM, Beck TW, Stout JR, Cramer JT (2009). Passive properties of the muscle-tendon unit: the influence of muscle cross-sectional area. Muscle Nerve.

[CR26] Schleip R, Duerselen L, Vleeming A, Naylor IL, Lehmann-Horn F, Zorn A, Jaeger H (2012). Strain hardening of fascia: static stretching of dense fibrous connective tissues can induce a temporary stiffness increase accompanied by enhanced matrix hydration. J Bodyw Mov Ther.

[CR27] Shih R, Wang Z, Heo M (2000). Lower limb skeletal muscle mass: development of dual-energy X-ray absorptiometry prediction model. J Appl Physiol.

[CR28] Thorton G, Shrive N, Franck C (2001). Altering ligament water content affects ligament pre-stress and creep behavior. J Orthop Res.

[CR29] Wattanapenpaiboon N, Lukito W, Strauss BJ, Hsu-Hage BH, Wahlqvist ML, Stroud DB (1998). Agreement of skinfold measurement and bioelectrical impedance analysis (BIA) methods with dual energy X-ray absorptiometry (DEXA) in estimating total body fat in Anglo-Celtic Australians. Int J Obes Relat Metab Disord.

